# Efficient simulation of neural development using shared memory parallelization

**DOI:** 10.3389/fninf.2023.1212384

**Published:** 2023-07-20

**Authors:** Erik De Schutter

**Affiliations:** ^1^Computational Neuroscience Unit, Okinawa Institute of Science and Technology, Okinawa, Japan; ^2^Department of Biomedical Sciences, University of Antwerp, Antwerpen, Belgium

**Keywords:** parallel algorithm, neural development, growth, migration, retraction, shared memory

## Abstract

The Neural Development Simulator, NeuroDevSim, is a Python module that simulates the most important aspects of brain development: morphological growth, migration, and pruning. It uses an agent-based modeling approach inherited from the NeuroMaC software. Each cycle has agents called fronts execute model-specific code. In the case of a growing dendritic or axonal front, this will be a choice between extension, branching, or growth termination. Somatic fronts can migrate to new positions and any front can be retracted to prune parts of neurons. Collision detection prevents new or migrating fronts from overlapping with existing ones. NeuroDevSim is a multi-core program that uses an innovative shared memory approach to achieve parallel processing without messaging. We demonstrate linear strong parallel scaling up to 96 cores for large models and have run these successfully on 128 cores. Most of the shared memory parallelism is achieved without memory locking. Instead, cores have only write privileges to private sections of arrays, while being able to read the entire shared array. Memory conflicts are avoided by a coding rule that allows only active fronts to use methods that need writing access. The exception is collision detection, which is needed to avoid the growth of physically overlapping structures. For collision detection, a memory-locking mechanism was necessary to control access to grid points that register the location of nearby fronts. A custom approach using a serialized lock broker was able to manage both read and write locking. NeuroDevSim allows easy modeling of most aspects of neural development for models simulating a few complex or thousands of simple neurons or a mixture of both.

**Code available at:**

https://github.com/CNS-OIST/NeuroDevSim.

## Introduction

Neural development is more than just growing another organ. During brain development, newly born neurons already establish initial connectivity. This enables infants to immediately start rewiring their networks during learning. Consequently, the experimental study on neural development is a large domain in neuroscience (Sanes et al., 2019). Conversely, rather little computational modeling of neural development has been published despite its clear importance in forming neural networks. Instead, even morphologically realistic network models are wired randomly using specific rules (Markram et al., 2015) instead of growing connectivity according to their development (Zubler et al., 2013). The most recent textbook on modeling neural development was published in 2003 (van Ooyen, 2003).

Several software packages are available to generate adult dendritic morphologies (Ascoli et al., 2001; Cuntz et al., 2010; Kanari et al., 2022) or connected networks of neurons with full dendritic morphologies (Koene et al., 2009), but these do not claim to simulate the actual developmental sequence. Recent time-lapse studies combined with modeling, mainly of Drosophila neurons, have greatly advanced our understanding of the developmental growth of single neurons (Yalgin et al., 2015; Baltruschat et al., 2020; Ferreira Castro et al., 2020; Palavalli et al., 2021; Shree et al., 2022; Stürner et al., 2022). But these studies ignore the effect of interaction and competition with nearby growing cells. To study such effects, we developed a computational framework called NeuroMaC (Neuronal Morphologies and Circuits) that grows multiple neuron morphologies simultaneously, emphasizing the importance of physical interaction between expanding neurons for dendritic development (Torben-Nielsen and De Schutter, 2014). Another simulator that explicitly models neural development based on intrinsic and extrinsic contextual factors was CX3D (Zubler and Douglas, 2009). It was used to build an impressive simulation of laminated cortex including a few complete neural morphologies (Zubler et al., 2013). CX3D is no longer supported and its more efficient, parallelized successor BioDynaMo (Breitwieser et al., 2022) is a general agent-based modeling platform that is not specific to neural development.

This paper presents a new Python software module for Neural Development Simulation, NeuroDevSim, that enables the simulation of most features of neural development with an emphasis on the interaction between new cells. Conceptually, it is a successor to the NeuroMaC software, but with a greatly improved model definition and computing speed. Software features and several innovative methods to enable pure shared memory parallelization will be described in section “Materials and methods”. The Results section covers mostly benchmarking of the parallel processing and tests of the shared memory implementation. For those readers familiar with NeuroMaC, comparisons will be made where appropriate.

NeuroDevSim was used to simulate a complex model of cerebellar development (Kato and De Schutter, 2023). This model simulates the first 14 postnatal days during which a massive migration of granule cells occurs from the top of the cerebellar cortex volume to the granular layer at the bottom, combined with the initial growth and partial retraction of Purkinje cell dendrites. The model contains more than 3000 neurons, including 48 Purkinje cells. We will refer to it in this paper to demonstrate some features of NeuroDevSim.

## Materials and methods

This paper describes NeuroDevSim version 1.0.1 which can be downloaded at https://github.com/CNS-OIST/NeuroDevSim; online documentation is available at https://cns-oist.github.io/NeuroDevSim/index.html. It runs on Python from version 3.6 onward on MacOS and Linux. NeuroDevSim does not work for Windows OS because it requires process forking to implement the shared memory parallelization.

Results were obtained using Python 3.9 on a MacBook Pro laptop with an M1 Max chip (eight performance cores) and 32 GB memory running macOS Monterey or on a PC with a 32-core AMD Ryzen Threadripper 3970X and 256 GB memory running Ubuntu Linux version 20.04 LTS. Run times were computed as the difference between the start and end times of the simulation_loop call. Run times for the Kato and De Schutter (2023) model measured the complete main code and were obtained on the high-performance computing cluster Deigo at OIST, using AMD Epyc 7702 CPUs with 128 cores and 512 GB memory. Memory use was tested on the 32-core AMD PC.

### Features replicated from NeuroMaC

The NeuroMaC software (Torben-Nielsen and De Schutter, 2014) focused on modeling the combined influence of internal and external factors on neuron growth, simulated as a sequence of growth *cycles*. The unit of a cycle is not defined and can correspond to any value in a range of approximately 1 to 100 h of real developmental time. Development was embodied in the concept of *fronts* that acted as independent agents simulating growth. A typical sequence was that a new front was made, either as an extension of a neurite or as part of a branching event. On the next cycle, this front would be active and execute a neuron-specific growth algorithm to decide whether an extension event, a branch event, or a growth-termination event should occur. The extension event resulted in the creation of one new front, the branching in the creation of two or more new fronts, and no fronts were created in the case of growth termination. The decision on which event occurred and the position of the new fronts depended on a combination of internal and external factors. Importantly, the software checked for front collisions; new fronts could not overlap with existing ones. At the end of a growth cycle, the original front was usually inactivated; it would not grow again. These growth events were stored as a tree structure, where the active front became the parent of one or more child fronts after an extension or branching event.

**FIGURE 1:**
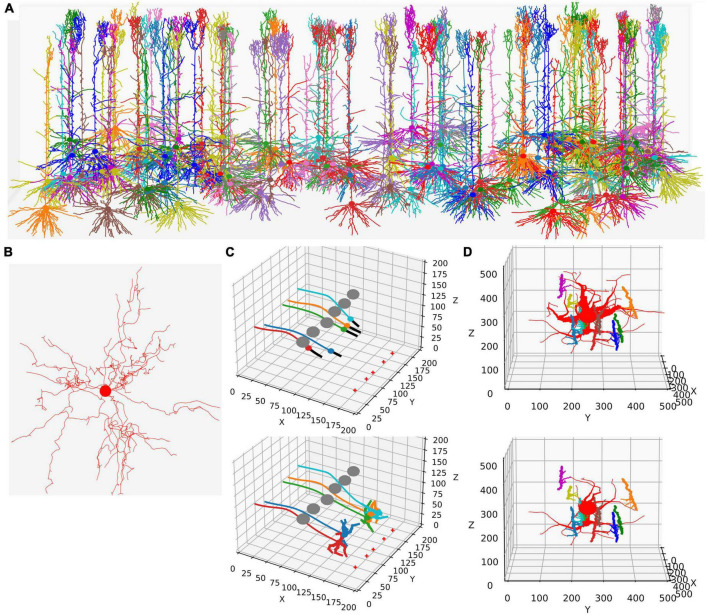
Examples of NeuroDevSim simulations. Panels **(A,B)** show results for previously published NeuroMaC models and are therefore plotted in the same style: dendrites as wire plots that do not show diameters (with the somata as small circles). Panels **(C,D)** are plotted as in the example of jupyter notebooks and represent the diameters correctly. All simulations shown are also available as Supplementary Movies. **(A)** The growth of a forest of 100 layer-5 pyramidal neurons (115 cycles of growth). **(B)** Dendrites and soma of a single alpha motor neuron (71 cycles of growth). **(C)** The migration of five somata with leading filipodium and trailing axons. Two images are shown of the simulation: during migration (cycle 20, top) and at the end of the simulation (cycle 34, bottom). At the bottom right of the simulation volume, the substrate is placed at five locations (red + symbols). The leading filipodia (black color) sense a stochastic representation of the local concentration based on an approximated diffusion of the substrate and grow toward increasing concentrations. The colored somata migrate along the filipodia and leave behind an axon of the same color. The simulation also contains six gray somata that do not grow but form an obstruction by repelling the growing filipodia. Notice how some of the axons curve around the obstructing gray somata; this shows the path followed by the soma that avoids the obstructions. When the filipodia sense very high substrate concentrations they retract, and migration stops. Note that though the substrate is dispersed along a line, the neurons clearly grew to only two locations and ended up forming clusters of two and three neurons, respectively. Once migration stops, each soma grows a few dendrites that avoid the dendrites of other neurons because no physical overlap is allowed. **(D)** Synapse formation leads to the retraction of dendritic branches with few synapses. The red neuron grows eight dendrites while axons with different colors grow around it from back to front (growth not shown). These axons extend small diameter side branches that make synapses on the red neuron dendrites. The top panel shows the result at cycle 89 before retraction. On cycle 90, five dendrites with less than five synapses are retracted instantaneously; the bottom panel shows the result at cycle 91 after retraction. The axon branches making synapses onto the dendrites are more visible in this bottom panel; note that some axon branches now lack a target because no axonal retraction was implemented.

**FIGURE 2:**
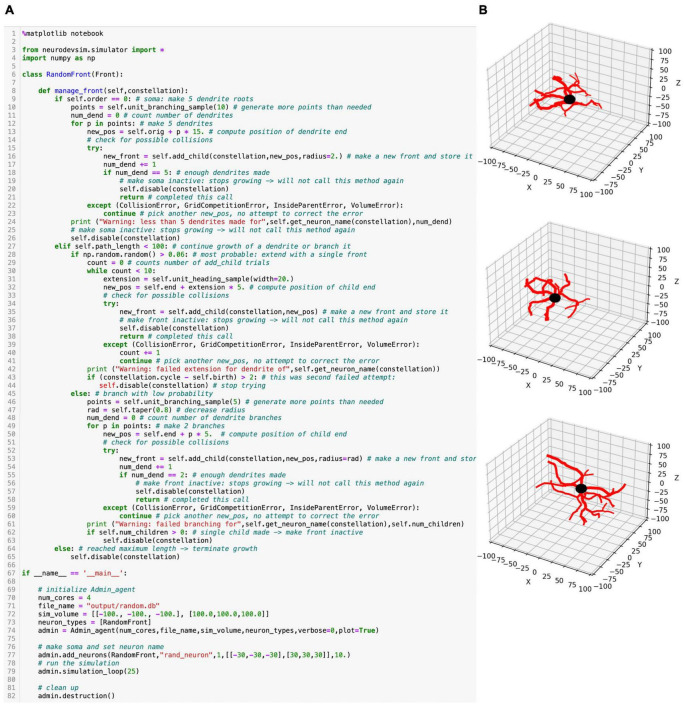
Random growth code example. **(A)** Python simulation script for a very simple NeuroDevSim model copied from a Jupyter notebook. Details of the code are explained in the text. **(B)** Three different runs of this script result in very different model results. In these plots, the soma is black (default), and the dendritic branches are red.

This functionality is replicated in NeuroDevSim using an updated model definition scheme explained in the next section. Modeling growth is illustrated by replicating the growth of a forest of 100 layer-5 pyramidal neurons [Figure 1A reproduces Figure 4 in Torben-Nielsen and De Schutter (2014), see also Supplementary Movie 1] and the growth of a single alpha motor neuron [Figure1B reproduces Figure 2 in Torben-Nielsen and De Schutter (2014), see also Supplementary Movie 2].

Like in NeuroMaC, the *substrate* can be used to provide chemical cues to growing fronts. The substrate can be initialized in the simulation volume (Figure 1C) or secreted by fronts. NeuroDevSim has built-in support to approximate the stochastic diffusion of the substrate (Figure 1C).

### New features of NeuroDevSim

In addition to growth, NeuroDevSim supports the simulation of migration and pruning, which are both important mechanisms in neural development. Migration is limited to somata before they grow dendrites and is restricted by collision detection. Two special features can make soma migration more biologically relevant: leading filopodia and trailing axons (Figure 1C). Leading filopodia function like growth cones, allowing for the exploration of the environment to find a proper migration path. NeuroDevSim allows for a single filipodium, with the soma migrating toward the position of the filipodium front that is a child of the soma. The soma can also have an axon, which is automatically extended as the soma migrates.

Pruning is implemented as front retraction; the retracted fronts are removed from the simulation. Entire branches can be retracted in a single cycle (Figure 1D) or slower pruning can be simulated with the removal of a single terminal front, possibly followed by additional single front retractions during subsequent cycles.

The impact of cellular migration and dendritic pruning on Purkinje dendrite development was demonstrated in the Kato and De Schutter (2023) model, with comparisons to control models where either mechanism was absent.

Wherein NeuroMaC synapses were randomly made structures that had only anatomical relevance, NeuroDevSim synapses are created between nearby pre- and post-synaptic fronts during growth (Figure 1D). They enable explicit simulation of the development of neural connectivity (Kato and De Schutter, 2023). Synapses do not occupy space. They have synaptic weights and as neurons have a firing rate, Hebbian (Hebb, 1949) or more complex synaptic plasticity rules (Cooper and Bear, 2012) can be simulated though at very slow time scales.

### NeuroDevSim model definition

NeuroMaC distributed the code and parameters of the Python simulation over several files, including a configuration file. However, NeuroDevSim is a Python module that enables the description of a complete simulation as a single Python script. This script can be run from a Jupyter notebook, if desired, with live graphics, or from the terminal. NeuroDevSim makes use of the Python Errors and Exceptions framework to deal with frequent problems like front collisions and simulation volume borders. An example of a simulation script for a very simple random growth model is shown in Figure 2A and some example results generated in a Jupyter notebook are shown in Figure 2B.

A NeuroDevSim script always contains two components: the definition of one or more Front subclasses that describe neuron-specific growth rules (lines 6–65 in Figure 2A) and a main program that creates an Admin_agent, populates the simulation volume with one or more somata, and runs the simulation (lines 67–82 in Figure 2A).

Like in NeuroMaC, the Admin_agent object manages the simulation and coordinates the different parallel processes that do the computing; this will be described in more detail in the next section. It also takes care of file output and, if specified, live graphics of the simulation as in this example. The first step in the main program is, therefore, the instantiation of an Admin_agent (line 74). Instantiation minimally requires specification of the number of computing processes to be used (num_cores = 4), the file name of the SQLite^1^ simulation database (file_name), the simulation volume in cartesian coordinates (sim_volume, in μm), and a list of all Front subclasses to be used (neuron_types). Because Admin_agent runs on its own core, this model uses five cores in total.

Next, a number of somata using one of the Front subclasses listed in neuron_types is created with the add_neurons method (line 77). In the example, one soma with the neuron name “rand_neuron” is made at a random location within a defined volume and with a radius of 10 μm. In case multiple somata are required, they can either be placed randomly in a volume or on a grid, or positions can be specified as a list of coordinates. Somata by default are spheres; other fronts are cylinders.

Now, the simulation can be run for a fixed number of cycles using the simulation_loop method (line 79), which can be called repeatedly so that, for example, additional neurons can be “born” at a later stage. Finally, at the end, the destruction method should be called to close down all parallel processes and complete the simulation database (line 82).

Additional methods can be called in the main program. Importantly, a previous simulation database can be imported with import_simulation immediately after Admin_agent instantiation; this can be used to run a series of simulations with a common initial condition. Other methods can deposit substrate (Figure 1C) or control storing of additional attributes in the simulation database.

The algorithms controlling growth are specified in the obligatory Front subclass method manage_front starting on line 8. Even for this simple RandomFront, the manage_front definition is fairly long. The manage_front method receives an obligatory parameter *constellation*, which is a complex data structure, with details hidden from the users, that contains all necessary data about the simulation and is passed to many other methods.

As manage_front has to deal with all possible RandomFront permutations during the entire simulation, it is usually structured as a set of nested if statements. When the method becomes too long, it can be subdivided by calling specific methods for each condition which improves the readability of the code.

The first if statement (line 9) distinguishes between the start of the simulation when only the soma exists, the growth cycle of dendrites (line 27), and the termination of growth (line 64). The first front created is the soma and this will generate the first call to manage_front at cycle 1. This condition is detected by if self.order = 0:, with only somata having a zero order of branching. The soma will grow the roots of five dendritic branches. To compute coordinates for these branches, 10 random points are generated around the soma with the unit_branching_sample method. More points than needed are generated because, in a more realistic simulation, many cells can be present, resulting in some locations already being occupied by other structures. Therefore, dendritic roots are grown by trial and error till either five roots are formed, or the method runs out of points. This is done by the loop starting on line 12, which contains a standard sequence to make new fronts.

New fronts will be children of self, the front for which manage_front is called. For cylindrical new fronts, their first coordinate *orig* will depend on the parent coordinate and only their second coordinate *end* needs to be specified as *new_pos*. The first fronts grown, dendrite roots, are a special case as they are connected to a somatic sphere, which has only one coordinate *orig*. *new_pos* is therefore computed relative to self.orig, to which a vector 15 μm long in the random direction *p* is added (line 13). Using this *new_pos*, the creation of a new child is tried with the front method add_child; note also that a new radius is specified as dendrites should have a smaller radius than the soma. If add_child is successful, it returns the new front and the number of roots *num_dend* is incremented. If *num_dend* reaches five, the soma is disabled because it should not grow further; this completes its call of the manage_front method (line 21).

Conversely, add_child can fail because of a number of errors. The prospective new child may overlap with an existing front, causing a CollisionError; a poorly written code may compute a *new_pos* which is inside the soma, causing an InsideParentError or which is outside of the simulation volume, causing a VolumeError; a GridCompetitionError explained in a later section may occur. All of these errors will just cause the code to try the next random point in *points* (lines 22–23). Alternatively, more complex responses to some of these errors can be programmed; for example, the solve_collision method may be called to try to overcome a CollisionError automatically. This use of the Python Errors and Exceptions framework results in a very flexible and readable way to deal with the problems that naturally arise when simulating growth in a crowded environment using parallel programming.

As mentioned before, the first part of the manage_front code is called once when only the soma is present. The next part, for the condition, elif self.path_length < 100: (line 27) will be called many times and codes most of the model growth. It first makes another decision using an if statement: whether to branch or just extend (line 28). This is done by drawing a random number using the numpy random library with a 94% probability of extension. For extension, a single front will be created. First, a pseudo-random direction is drawn that falls within a 20-degree cone around the current heading with the unit_heading_sample method (line 31). This is then added to the self.end coordinate of the parent cylinder with a length of 5 μm. The add_child method is tried again and errors are caught. If add_child is successful, the parent is disabled and manage_front returns (line 38), otherwise the *while* loop (line 30) is continued, and a new pseudo-random direction is drawn. This is repeated till add_child is successful, which for this simple model will always work within a few trials.

With a 6% probability, a branching event will take place. This is done similarly to the code for making dendritic roots at the soma; the difference is that now, only two branches need to be made, so fewer random points are requested, and a new smaller radius is computed relative to the radius of the parent with the taper method (lines 45–63).

Finally, when self.path_length, the accumulated distance between the *end* coordinate of self and the soma, is longer or equal to 100 μm, growth terminates: the front is disabled without calling add_child (lines 64–65).

This simple code shows the simplicity of programming robust growth in NeuroDevSim. The full description of the simple random growth model is 65 lines of code. For comparison, the code for the realistic single alpha motor neuron model (Figure 1B) is not much longer at 81 lines with the main differences being branch order dependent control over branching probability and different lengths of individual branches. In contrast, the code for the layer 5 pyramidal neuron forest (Figure 1A) is more complex, with many additional methods, and comprises 372 lines. This is because four different types of dendrites are grown: basal, apical, oblique, and tuft dendrites and the growth of the latter ones is cortical layer-dependent. In addition, repulsion by nearby dendrites from the same neuron is simulated.

The example in Figure 2 uses the add_child method only, other NeuroDevSim features are accessible with the front migrate_soma (used in Figure 1C), retract, and retract_branch (used in Figure 1D) methods. All of these methods and options to have environmental control of growth are demonstrated in numerous example notebooks that come with the NeuroDevSim distribution.

### Collision detection

A major computing cost is the detection of overlap between existing fronts and new fronts that add_child tries to create; such a structural overlap is called a collision. NeuroDevSim uses the same algorithm as NeuroMaC to detect collisions but in a more scalable implementation. Cylindrical fronts are represented by the line segments that form their central long axes and the distance between the closest points on each line segment is computed using the dist3D_Segment_to_Segment algorithm.^2^ If this distance is smaller than the sum of the radii of the two fronts, a collision is detected and add_child returns a CollisionError.

Obviously, the collision detection should occur only with existing fronts that are spatially close to the proposed new one as computing distances to all existing fronts rapidly become very time-consuming. In NeuroMaC, spatial subvolumes partitioned fronts according to their point of origin, and collision detection was only performed with fronts in the same subvolume. But as subvolumes could be quite large, they often included distant fronts.

NeuroDevSim uses a grid to test collisions with close-by fronts only. The 3D grid spans the entire simulation volume and grid points are separated by a predefined distance grid_step along all axes, with a default of 20 μm. Each front is allocated to at least one grid point; if it is large or positioned at about an equal distance between multiple grid points, it will be allocated to additional grid points. Similarly, the proposed new front is matched to one or more grid points, which, in this study, are called G. Collision detection involves testing all fronts allocated to G and their immediate neighboring grid points, with 26 neighbors for each grid point. The standard approach is to raise a CollisionError when a first collision is found, with the CollisionError returning this first colliding front. It is possible to force a complete search and obtain a list of all colliding fronts, but this slows down the simulator.

### NeuroDevSim code overview

NeuroDevSim uses a very different way to parallelize the simulation than NeuroMaC did. NeuroMaC subdivided the simulation volume into subvolumes, with each spatial subvolume being managed by a different computing core. In addition, a dedicated core ran Admin_agent and all front updates had to be communicated to the Admin_agent core. Communication between cores was messaging-based and implemented with the ZeroMQ^3^ library. This approach to parallelism was simple but created several problems. First, if the number of fronts diverged between subvolumes, the simulation became unbalanced with some cores finishing much faster than others. While unbalancing sometimes could be reduced by choosing the right subvolume division scheme, for example, not subdividing the vertical axis in the pyramidal neuron forest example (Figure 1A), it led to a limited scaling behavior for many models. Moreover, dealing with fronts that originate in one subvolume but end in another one required communication between cores that also had to implement the eventuality that such a new front collided with existing ones and had to be discarded. A robust simulation of front migration and retraction across subvolumes was even more challenging.

**FIGURE 3:**
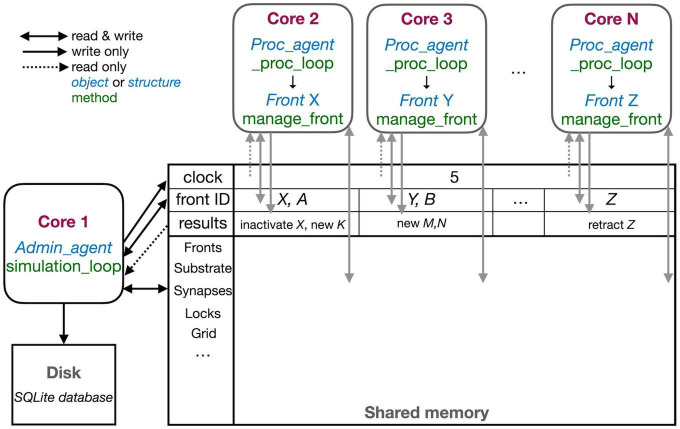
The schematic representation of the parallel workflow using shared memory. The default situation, without live plotting, is shown. The simulation is controlled by Admin_agent that runs on Core 1 (left). Its interaction with processing cores during the execution of simulation_loop is shown. Admin_agent controls the clock that synchronizes all cores, which is available in shared memory. When a new simulation starts, Admin_agent puts the front IDs of fronts to be processed in dedicated areas of shared memory for each processing Core 2-N **(top)**. The latter run _proc_loop that fetches the front corresponding to the ID executes its manage_front method and puts any new structures in the results array in shared memory as their ID plus a symbol. Admin_agent then fetches these results to store them in the database (**bottom** left) and to update its list of active fronts for the next cycle.

Conversely, NeuroDevSim does not use spatial subdivision, but instead, dynamically allocates fronts to different cores for computation. This allows for a close to perfect balance between the computing cores. Moreover, instead of messaging, it uses a shared memory approach that gives each core access to all data and that is also used to schedule jobs for computing cores. The use of shared memory is shown schematically in Figure 3. In this section, the general flow of simulating a cycle was described. The next section will consider parallelization using only shared memory in more detail.

The different cores running Admin_agent and, for processing, Proc_agent are not synchronized except at the beginning of a cycle. Admin_agent controls the cycle, the value of which is stored in shared memory as a clock so that it can be read by all cores. In contrast with Python practice, everything is numbered from one because index 0 indicates an unused or empty index in NeuroDevSim. In most arrays, index 0 is therefore never used and Admin_agent runs on Core 1.

Each of the processing cores, numbered Core 2 to N in Figure 3, continuously runs a simple loop _proc_loop. It reads the current value of the clock in shared memory, and when the cycle is incremented, it starts fetching front IDs from a private section in shared memory. A front ID is an object encoding indexes into the Fronts array in shared memory. The front is accessed, the ID is cleared to signal Admin_agent that front processing has started, and the manage_front method is called. For example, in Figure 3, Core 2 receives an instruction to start processing front *X* and it will call the manage_front method of *X*. If new fronts or other structures like substrate or synapses are created during manage_front, they are immediately stored in a results array in shared memory; in the Core 2 example, a front *K* was created. The outcome of manage_front is encoded in the private results section of shared memory as a series of front IDs plus symbols specifying what happened to each front. Note that run times can be very different for each front; this depends on front-specific rules and on whether collisions happen or not. In the example shown in Figure 2, the soma front will take more computing time to create five dendritic roots than dendrite fronts that make only one to two children. Also, checking for collisions will be much faster when fewer fronts are present compared to more crowded areas of the simulation volume. _proc_loop keeps fetching front IDs till it receives a unique one that signals the end of the cycle. It then performs minimal maintenance and goes into intermittent sleeping mode till the next cycle starts.

Admin_agent controls the cycle, incrementing it from zero to one at the start of the simulation. As mentioned, this signals processing cores to start computing. One task of Admin_agent is to supply processing cores with front IDs as fast as possible, ensuring that each core is kept busy. As long as many active fronts need to be computed, it will provide each computing core with two front IDs to make sure that the core can run continuously, as shown for Cores 2 and 3 in Figure 3. However, at the end of the cycle, when only a few fronts remain to compute, only a single front ID is provided (Core N in Figure 3). Once all active fronts have been scheduled, the unique end-of-cycle ID is sent to the cores. At the end of the cycle, some processing cores may be idle while others are computing the last fronts for this cycle. The order in which fronts are computed is managed dynamically to avoid grid competition as will be explained in the next section; in general, growing fronts are computed first, followed by migrating ones, and then other active fronts. Importantly, the scheduling of front processing to specific computing cores tends to vary for every run of the model because how soon a computing core finishes depends on its working condition, which is beyond the control of NeuroDevSim. Optimal front scheduling requires the user to actively control the growth/migration/active status of fronts. In many cases, this just requires calling the disable method at proper times, as shown in Figure 2, but a more complex code may be required.

Besides scheduling fronts to be computed by processing cores, Admin_agent also performs file storage. It uses the information provided by the processing core in results shared memory to store information about new fronts and other new structures, soma migration to new positions, and front retractions in the simulation database. Finally, if simulation_loop is not finished, it updates its lists of growing, migrating, and active fronts to prepare for the next cycle and increments the cycle.

In the case of interactive plotting in Jupyter notebooks, a second admin core is used and the functionality of Admin_agent is split over two cores to improve processing speed. The main admin core still sets the cycle and processes the results returned by processing cores both for file storage and plotting. It also starts the second admin core which only performs front scheduling.

### Parallel computing based uniquely on shared memory

NeuroDevSim uses the multiprocessing sharedctypes Python libraries to provide parallelism. All shared data is stored in RawArrays, plus the clock as a RawValue. These shared arrays are created early during Admin_agent initialization before the different processing cores are forked. All shared objects, like fronts, substrates, and synapses, are defined as ctypes Structures. As a consequence, their attributes are typed and because Structures are fixed size, instant-specific attributes are not supported. However, in the specification of a Front subclass, the user can define additional attributes that will be present in every instance of the subclass (not shown in this paper).

Because all entries in arrays are fixed size, a custom code is needed to deal with Python lists. For short lists, like the list containing the indices of each child of a front, linked lists are used.^4^ For longer lists, like those used by the 3D grid, linked blocks are used. These are accessed by an index to a block of predefined size in the array. Empty spots in the array block can be detected by their zero values and are typically filled from the beginning to the end. The last entry of each block is an index to the next block in the same array if needed.

Passing simulation data through shared memory is very efficient and simple as long as it is read-only. The challenge is to write data in such a way that different cores do not write to the same memory location at the same time. Python provides a Lock mechanism, but this does not scale well for the large, shared arrays used by NeuroDevSim. Instead, specific solutions were developed that avoid locking as much as possible.

**FIGURE 4:**
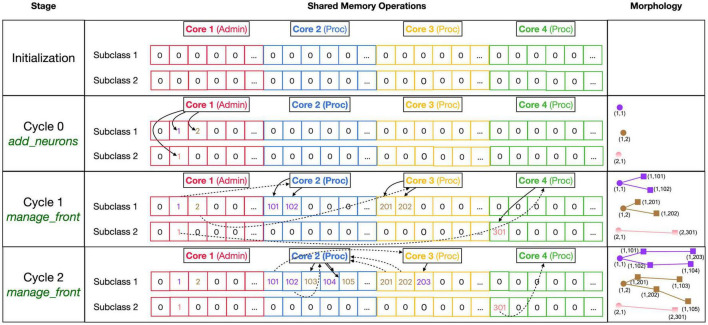
Division of _fronts shared memory array to prevent writing conflicts. The array, shown in the center part, is two-dimensional: each front Subclass has its own row, and each Core has its own color-coded private section of columns spanning all Subclass rows. The changes in array contents for four different stages, indicated in the **left** part, are shown. The **right** part of the figure shows schematic 2D representations of the morphology of three neurons, two of Subclass 1 and a single of Subclass 2. The fronts are color-coded with circles for spherical somata and squares showing the end of cylindrical fronts. The numbers are front IDs, consisting of (index of subclass array and index in subclass array). In the center, sections of the shared _fronts array are shown. Zero indicates an empty location and locations that contain a front ID show their index in the corresponding neuron color. Cores can write only to the color-coded part allocated to them; full arrows indicate how they write IDs of new fronts at specific indices during each stage. Broken arrows show how Cores can read front IDs anywhere in the array so that they can call the corresponding manage_front method to make new fronts. See text for more details.

The main approach to avoid locking is to subdivide all main data arrays in shared memory into sections so that processing cores write data to their private section only. Figure 4 shows the shared data structure containing fronts for a fictional example (no code shown) in detail; similar approaches are used to store substrate, synapses, etc. The _fronts array is two-dimensional; it is first subdivided by a front subclass, with a row for each of the two subclasses (defined in neuron_types) in Figure 4. Because all structures are fixed size, and Front subclasses may have different sizes if additional attributes are defined, each subclass is stored in a separate array. These arrays are then subdivided into num_procs + 1 equal private section (colored differently in Figure 4). All cores can read anywhere from these arrays but only write to their private section which contains 100 entries in this example. Each core maintains counters for the next free index in the private section of each of the subclass-specific arrays. Whenever a core makes a new front, it checks whether the counter is larger than its maximum index and generates an OverflowError if that is the case. Otherwise, it stores the front at the counter location and increments the counter.

Figure 4 shows the sequence of the creation of three neurons, two belonging to subclass 1 and a single one for subclass 2, during initiation and the first two cycles of simulation of a simple case with just three processing cores (Cores 2–4). The right column shows a schematic representation of their morphologies in 2D, with circles representing spherical somata and squares representing the end coordinate of cylindrical fronts. An add_neurons call during initialization (cycle 0) results in the creation of three somata, which will all be stored in the private section of Core 1 which runs Admin_agent, each soma in the correct subclass array. Their front IDs are (index of subclass array and index in subclass array) and are shown below the corresponding circles in the Morphology column: (1,1), (1,2), and (2,1). Note that the private _fronts array section of Core 1 will contain somata only.

During cycle 1, each of these somata will be processed by different cores: soma (1,1) of the purple neuron is processed by Core 2, soma (1,2) of the brown neuron by Core 3, and soma (2,1) of the pink neuron by Core 4. For each soma, the corresponding core calls its manage_front method, which in turn calls add_child twice for the purple and brown neurons (subclass 1) and once for the pink neuron (subclass 2). Because the purple neuron was allocated to Core 2, its children are stored in its private section and receive IDs (1,101) and (1,102). The brown neuron acts similarly on Core 3 with IDs (1,201) and (1,202) and the pink one on Core 4 with ID (2,301). Next, during cycle 2, the children created during the previous cycle will be processed by different cores. Now, the order of scheduling fronts to cores becomes relevant. Figure 4 shows a possible sequence, but this could be different in another run of the same model as it depends on the order in which new fronts were processed by Admin_agent during the previous cycle. The first front to get scheduled is the brown front (1,201) for Core 2; this makes a single child (1,103). The next is the purple front (1,101) on Core 3 making a single child (1,203), followed by the pink front (2,301) on Core 4 which does not make a child. The processing of (1,101) on Core 3 and (2,301) on Core 4 takes more time so that they are not available for the remaining two fronts which are, therefore, both processed on Core 2: purple front (1,102) makes child (1,104) followed by the brown front (1,202) making its child (1,105).

Note that the entry of the parent, child, and soma indices in new fronts never causes writing conflicts. The parent and soma indices are set for the newly created fronts that are stored in the core’s private section. The child indices are set for the parent front that may be located in another private section [e.g., front (1,101) making the child (1,203)], but the parent is the front calling manage_front as self, and during this call, it is guaranteed that no other cores will try to change its attributes. Users are instructed not to change front attributes other than those of self, except under well-defined exceptional circumstances. To enforce this rule, methods like add_child, migrate_soma, retract, etc., raise a NotSelfError when not called by self.

The approach described combines simplicity with robustness for safely storing new fronts but results in wasteful use of memory, e.g., in Figure 4, most of the subclass 2 array is not used. Because the private sections of the arrays must be sufficiently large, the overall size of the _fronts array is large and it usually contains many empty locations. However, as this needs to be allocated only once in shared memory (and not separately for every core), the overall memory use is modest for modern architectures (see section “Results”).

Unfortunately, this approach does not work when fronts are retracted. For simplicity, they are not deleted from the _fronts array so that computing cores do not need to fill empty spaces. Instead, they are flagged as retracted and removed from their parent’s list of children. The latter action could cause memory writing conflicts when a complete branch is retracted because retracting fronts at the tip of the branch may simultaneously be calling manage_front on another core. Therefore, as it is assumed that front retractions are rare events, the memory updates needed for front retraction are done in a serial fashion by Admin_agent at the end of each cycle.

**FIGURE 5:**
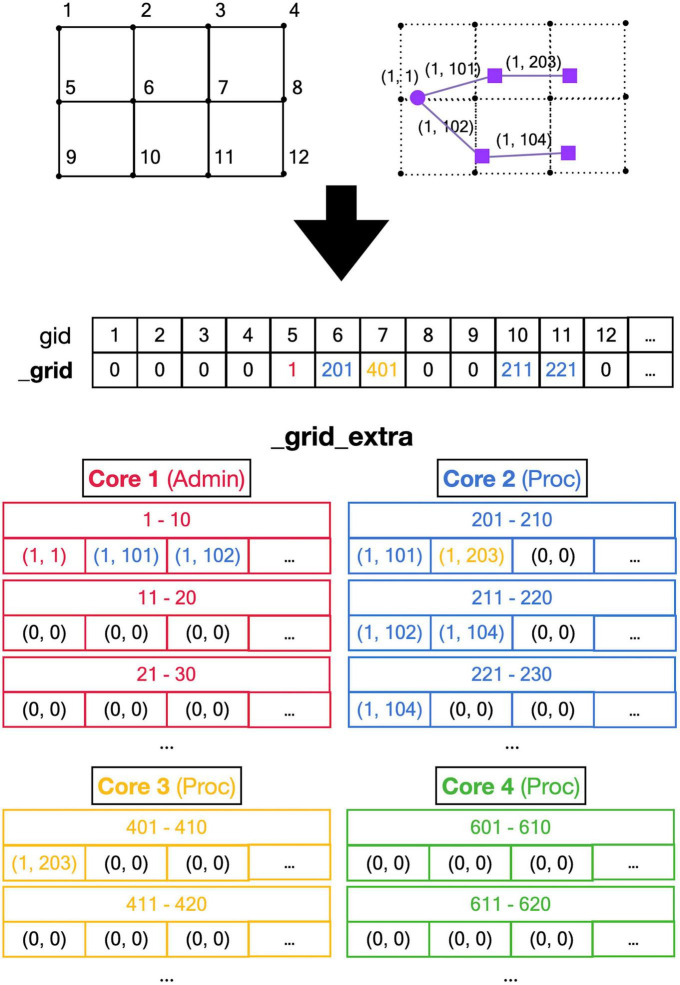
Registering front coordinates in a grid. **Top**: A 2D representation of different grid locations in space; in actual code, the grid is 3D. The left shows the index *gid* associated with each location and the right shows the purple neuron from Figure 4 mapped onto the grid with front IDs. **Mid**: _grid shared array is indexed by *gid*. If no fronts are located at a grid point, the entry is zero, otherwise, it is an index into the _grid_extra array. Used indices are color-coded for the Core that made the entry (see Figure 4). **Bottom**: the representation of _grid_extra shared array. This linear array is subdivided into private sections for each Core, color-coded. When a Core needs to register a coordinate at a grid location that is empty, it will allocate a new block (default size 10) in its private section of _grid_extra. For example, when Admin_agent (Core 1) makes soma (1,1) during the add_neurons call, it allocates a block for _grid point 5 starting at index 1 in _grid_extra and writes the front ID (color-coded for the Core that made the entry) at that index. As shown in Figure 4, Core 2 makes two cylindrical child fronts for (1,1) during cycle 1. Because the origin coordinates of these fronts (1,101) and (1,102) are located close to _grid point 5, they are entered at the next free indices 2 and 3 in the red block in _grid_extra. The end coordinates of (1,101) and (1,102) are close to _grid points 6 and 10, respectively. So, Core 2 allocates two new blocks in its private section of _grid_extra, starting at indices 201 and 211, and enters the corresponding front IDs. During cycle 2 front (1,203) is created by Core 3, which allocates a new _grid_extra block at index 401 for _grid point 7, and front (104) is created by Core 2, which allocates a new _grid_extra block at index 221 for _grid point 11. The origin coordinates of these two fronts are added to previously allocated blocks at indices 202 and 212. When blocks fill up, for example, when an 11th front needs to be allocated to _grid point 5 using indices 1–10 in _grid_extra, a new block needs to be allocated in _grid_extra (not shown), for example, at indices 411–420 (Core 3 making the 11th front). This new block is linked to the existing one by changing the entry at index 10 to (–4,110), the front ID that used to reside at index 10 is moved to index 411, and the 11th front is registered at index 412.

A major challenge caused by the use of shared memory is the updating of the 3D grid used to detect collisions (Figure 5, top). The grid is mapped onto a one-dimensional integer _grid array so that the x, y, z position of grid points uniquely determine an index called *gid*, larger than zero, in this array (Figure 5, mid). Entries in _grid are zero for points with no fronts or an index to linked blocks of default size 10 in a _grid_extra array containing front IDs (Figure 5, bottom). New blocks in _grid_extra are allocated to core-specific private sections, but once allocated, all cores can write to them. At present, new grid entries are added by traversing the _grid_extra block till an empty spot is found. When a grid entry needs to be removed after front retraction, it is replaced by the last entry in _grid_extra. Both _grid and _grid_extra are read and updated frequently by all computing cores, so preventing simultaneous access by different cores has to be controlled carefully.

**FIGURE 6:**
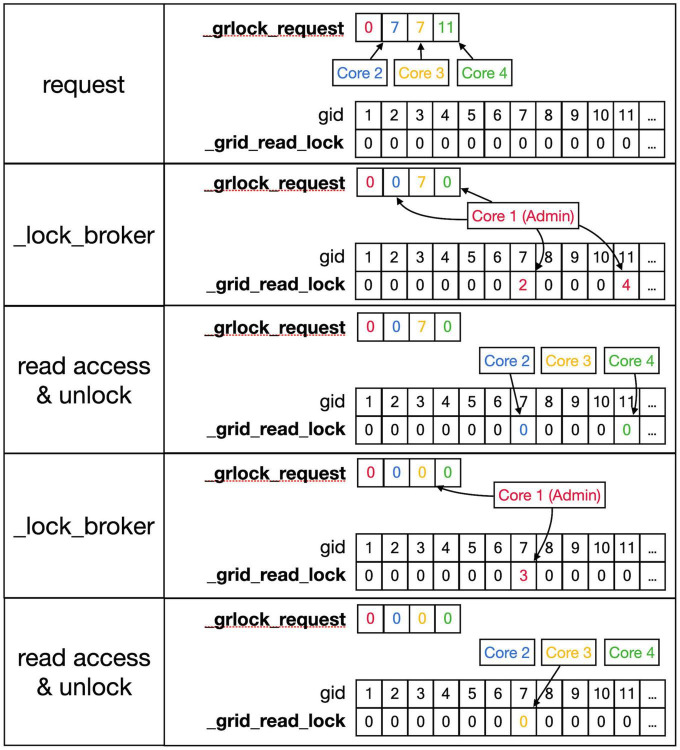
Sequence to lock _grid arrays for reading. This shows a series of events resulting in giving a Core read access to the _grid and _grid_extra shared arrays. **Top** row: As described in the text, Cores write *gid*, an index into the _grid array (see Figure 5) into their private location in the shared _grlock_request array, *gids* are color-coded for Cores. Cores 2 and 3 both request access to *gid* 7 and Core 4 requests access to *gid* 11. The _grid_read_lock shared array indicates the locking status for each *gid* value, with zero meaning no lock. Colored entries in this array will be coded for the Core that did the writing. Second row: _lock_broker, a method running serially on Core 1, reads entries in _grlock_request and locks the corresponding *gid* locations in _grid_read_lock if they were zero by entering the number of the core. It also resets the request in _grlock_request to zero. Because both Cores 2 and 3 requested access to *gid* 7, only Core 2 is giving a lock and Core 3 has to wait. Third row: Cores 2 and 4 notice that their indices appeared in _grid_read_lock for the *gid* entries they requested (7 and 11, respectively) and they now read the information in the grid arrays; when done, they clear the lock in _grid_read_lock by setting it back to zero. The request of Core 3 in _grlock_request remains active. Fourth row: _lock_broker can now execute the request of Core 3 and locks *gid* location 7 in _grid_read_lock for Core 3, again resetting _grlock_request. **Bottom** row: Core 3 can now perform a grid read at *gid* 7 and unlocks _grlock_request.

**FIGURE 7:**
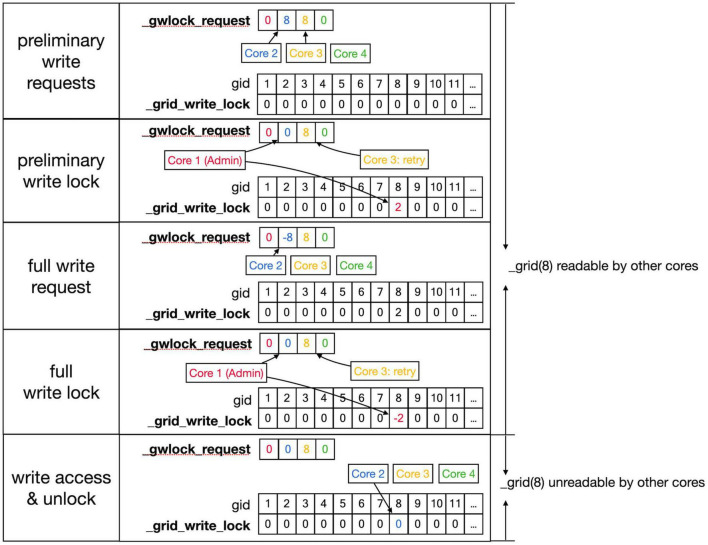
Sequence to lock grid arrays for writing. Writing access to the _grid and _grid_extra shared arrays uses shared arrays similar to those shown in Figure 6 for reading, called _gwlock_request and _grid_write_lock. Writing locks occur in two stages: the preliminary stage indicates that a Core may want to write to this location in the near future. The preliminary write locks are used for front scheduling by Admin_agent and during collision detection. During the preliminary write lock of a *gid*, read locks can be obtained from it (see Figure 6). **Top** row: Core 2 and 3 both request preliminary write access to *gid* 8 in _gwlock_request. Second row: _lock_broker detects these requests and gives Core 2 preliminary write access by writing its index at *gid* 8 in _grid_write_lock. It also resets the request of Core 2, while that of Core 3 remains. Third row: sometime later Core 2 requests full write access by entering in _gwlock_request a negative *gid* value (–8) for the location it has already preliminary locked. Instead, if Core 2 notices that it does not require writing, it will immediately remove the preliminary lock at *gid* 8 (not shown). The preliminary write request of Core 3 is still waiting. Fourth row: _lock_broker gives write lock access by entering the negative index of the requesting Core (–2) in _grid_write_lock and clears _gwlock_request. **Bottom** row: noticing the –2 value at gid location 8 in _grid_write_lock, Core 2 now performs its writing action upon the grid arrays. During this write action, no other cores are allowed read or write access to the corresponding memory locations. When done, Core 2 clears the lock. If the entire sequence described above took less than a second, Core 3 will now receive preliminary write access. If not, Core 3 will raise a GridCompetitionError.

Conflicts between cores are avoided by a layered locking mechanism illustrated in Figures 6, 7. Each core is identified by its number, called *pid*. They have a private location in the _grlock_request and in _gwlock_request arrays in shared memory, which by default contain zeros. When they need access to the 3D grid, they put the corresponding *gid* value in the private location of one of these request arrays and wait till the lock is obtained or a predetermined period has passed. If a lock cannot be achieved in time, a GridCompetitionError is raised. The request arrays are monitored by the _lock_broker method that is run intermittently by Admin_Agent. Because _lock_broker is the only method that is allowed to set grid locks, locking is a serial process.

As shown in Figures 6, 7, _lock_broker uses shared memory _grid_read_lock and _grid_write_lock arrays to lock specific access to gid entries in _grid. When _lock_broker detects a request, it checks whether that gid is currently locked for the read (Figure 6) or write (Figure 7) action requested. If the lock value was zero, it was unlocked and will be locked by setting it to the pid of the requesting Core; _lock_broker also clears the request in _grlock_request or _gwlock_request by setting it back to zero. The requesting Core monitors the value of _grid_read_lock or _grid_write_lock at index gid and starts accessing the grid once it is set to its unique pid value. When it finishes accessing the grid it resets the lock to zero. Note that cores can only access their private sections of the _grlock_request and _gwlock_request arrays, but they can access any index in the _grid_read_lock and _grid_write_lock arrays. Conversely, _lock_broker has access to all data structures shown.

The locking sequence described applies to locking for read-only access (Figure 6); for writing, it is more complicated (Figure 7). A preliminary write lock is obtained first, with a positive gid value in _gwlock_request array, followed by a full write lock, with a negative gid value in _gwlock_request array before the actual writing starts. Read locks can be issued for *gid* that are under preliminary write lock, but not for *gid* that are under full write lock because the memory content is unreliable during writing. The preliminary write lock is used to prevent different processing cores from trying to perform actions that could lead to attempted updates of identical grid points. During an add_child sequence, the grid points to be occupied by the prospective new front are computed and preliminary write locks are requested. If a lock cannot be obtained within 1 s, a GridCompetitionError is raised; this is, in general, due to multiple cores trying to grow in the same area of the simulation space. To reduce the likelihood of this happening, Admin_agent sets preliminary write locks for all grid points associated with a scheduled front and only schedules fronts for processing that do not occupy already write-locked grid points. The main customer of grid locking is the _test_collision method that checks whether proposed new fronts collide with existing ones. It is highly optimized to, if necessary, try obtaining needed preliminary write locks or read locks repeatedly to reduce the likelihood of GridCompetitionError. If the new front passes the collision check, it will be added to the grid, and for that, the full write lock has to be obtained first, but this lock is maintained only very briefly.

It is up to the user to determine how to deal with a GridCompetitionError. Often the add_child method causing it is just repeated in the hope that other cores will release their lock on the grid points needed. But this should be tried only a few times to avoid wasting time on persistent GridCompetitionErrors. In the case of simulations with many cycles, a good alternative is to abort the add_child call and try again during the next cycle.

## Results

The results section of this paper focuses on the performance of the software. First, performance on a performant laptop is described because many small models can be run effectively on such accessible hardware. Next, extensive benchmarking is shown, including comparisons with NeuroMaC. These benchmarks were run on a dedicated PC with a 32-core CPU. Benchmarking results for the Kato and De Schutter (2023) model on a computing cluster are also shown.

### Running NeuroDevSim on a laptop

For simple simulations, like those in the notebooks in the “examples” directory, run time is quite short on a modern laptop. For example, approximate run times on four cores on a laptop with several other apps open are, respectively, 3.6–6.0, 2.3–3.2, and 23–52 s for the models shown in Figures 1B–D with live plotting enabled (*n* = 5). Plotting strongly increases run times; without plotting, run times become 0.5–0.9, 0.3–1.0, and 1.3–1.9 s, respectively.

**FIGURE 8:**
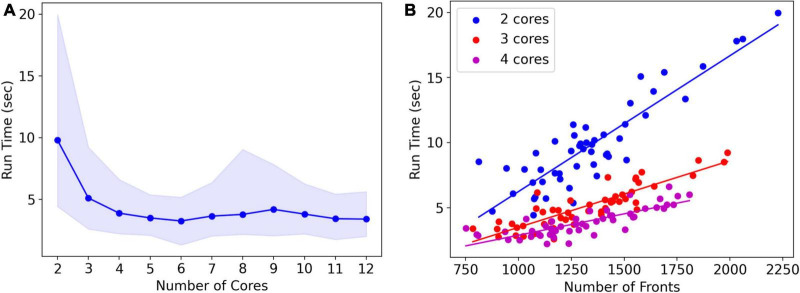
Run times of L5 pyramidal neuron model on a MacBook Pro laptop. **(A)** Mean run time in seconds for 120 cycles of simulation vs. the number of cores used (*n* = 50). Shaded area plots minimum and maximum run times, showing a large variance in run times that decreases with the number of cores used. Note that the minimum number of cores is 2: one for *Admin_agent* and one for *Proc_agent*, resulting in a serial simulation. All other core numbers are for parallel simulation, with (number of cores –1) processing cores. Run times rapidly decrease up to five processing cores (six cores in total); parallel simulation is less efficient for more cores due to the small model size. The hardware had eight high-performance cores. **(B)** A single trial run time in seconds vs. the number of fronts generated by the simulation for different numbers of cores used. The variability of run times was strongly correlated with the number of fronts generated. Correlation coefficients were 0.93, 0.93, and 0.82 for two, three, and four cores. For all cases in panel **(A)**, the correlation was highly significant with *p* < 0.0001.

**FIGURE 9:**
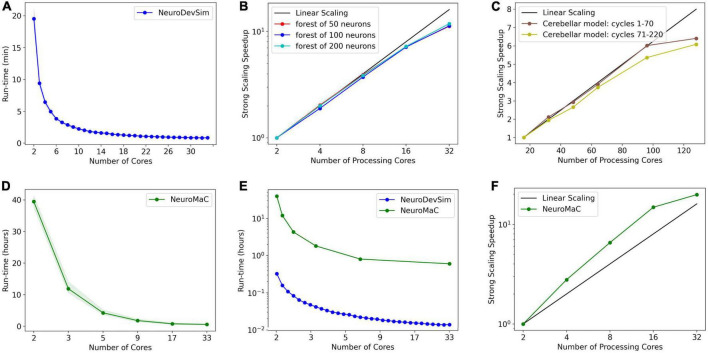
Run times and strong scaling of more complex models using NeuroDevSim **(A–C)** or NeuroMaC **(D–F)**. Data was collected on AMD Ryzen Threadripper 32-core **(A,B,D-F)** or AMD Epyc 128-core CPUs **(C)**. Run times are shown relative to all cores used, while parallel strong scaling is shown relative to processing cores only (one core less). **(A)** Mean run time in minutes for 115 cycles of NeuroDevSim simulation of the L5 pyramidal forest of 100 neurons vs. the number of cores used (*n* = 10). **(B)** Strong scaling of NeuroDevSim for three different forest sizes of 50, 100, and 200 neurons, respectively. Note that scaling improves with forest size though differences between 50- and 100-neuron forests are small. Linear scaling corresponds to optimal parallelization relative to the 2-core reference (*n* = 10). **(C)** Strong scaling for the Kato and De Schutter (2023) model relative to the 16-core reference. Data for 32, 48, 64, 96, and 128 cores are shown. The first 70 cycles show close to optimal scaling for up to 96 cores; scaling for separate simulations of later cycles is influenced by additional serial processing (see text) (*n* = 5). **(D)** Mean run time in hours for 115 cycles of NeuroMaC simulation of the L5 pyramidal forest of 100 neurons vs. the number of cores used (*n* = 10). Note that the available number of processing cores combinations is limited; they correspond to spatial (x, y, z) subdivisions of (1,1,1), (2,1,1), (4,1,1), (8,1,1), (8,2,1), and (16,2,1), respectively. Other subdivision schemes, like (4,2,1) and (4,4,1), were also tried but gave similar results. Shaded area plots’ minimum and maximum run times. **(E)** Comparison of run times of NeuroDevSim and NeuroMaC simulations of the 100-neuron forest. Note the semi-log scale. **(F)** Strong scaling of NeuroMaC for the simulations shown in **(C)**.

Note that run times are quite variable. A systematic analysis of run times and their dependence on the number of cores used was done for the L5 pyramidal neuron model, simulating just one cell (Figure 8A). As expected, mean run times rapidly decrease with an increasing number of cores, speeding up from 9.8 s for serial simulation (two cores) to 3.3 s for parallel simulation with six cores. Beyond six cores, there is no further speed-up, instead, run times increase slightly. This behavior is due to the small model size, see Figure 9, where much larger models are benchmarked.

Run times were quite variable from simulation to simulation, shown by the shaded area in Figure 8A. The variability of run times was strongly correlated with the number of fronts generated during the simulation (Figure 8B). Because branching events are probabilistic in this model, the number of branching events varies between simulation runs resulting in small models with few branch points or large ones with many branch points, depending on the random number sequence. As the number of cores increases, the highly variable number of fronts generated on each core is averaged by a larger n, resulting in a lower variance of the total number of fronts and somewhat fewer variable run times.

Importantly, each parallel NeuroDevSim simulation is always unique; it cannot be reproduced for reasons described in section “Materials and methods.” In fact, the random seed which can be specified during Admin_agent instantiation only affects the random placement of somata by the add_neurons method. To obtain a reproducible simulation, NeuroDevSim has to be run in serial mode with only a single processing core (two cores in total), only then will the random seed also affect the total simulation outcome. The user has the choice between slow, reproducible serial simulation, or fast but unreproducible, parallel simulation. For model debugging purposes, serial simulation may be preferred, but otherwise, the speed advantages of parallel simulation are obvious.

### Memory use

NeuroDevSim default settings allow for simulations with up to 20,000 fronts, which is suitable for modeling the growth of hundreds of small neurons or a few large ones. Default memory use is 96 MB for simulations using a few cores; this increases to up to 221 MB for simulations using more than 10 cores. For larger simulations like the forest of 100 pyramidal neurons simulation shown in Figure 1A which is used for benchmarking, two array sizes had to be increased to accommodate up to 30,000 fronts. This increased memory use to 209 MB for 2-core simulations, 1.1 GB for 10-core simulations, and 1.7 GB for 25 to 32-core simulations.

### Performance benchmarks

NeuroDevSim run times for different model sizes were evaluated on a dedicated PC with a 32-core CPU and compared to similar benchmarks for NeuroMaC. For these benchmarks, run times of 10 simulations of 115 cycles each of the forest of layer 5 pyramidal neurons simulation shown in Figure 1A were averaged (Figure 9). The number of fronts made by NeuroMaC was also highly variable and scaled with run time. Minimum–maximum range over all simulations was slightly smaller for NeuroMaC (126,655–137,083 fronts) than for NeuroDevSim (124,013–138,997 fronts) but because fewer NeuroMaC simulations were run, this small difference is probably due to under-sampling.

For the standard simulation of a forest of 100 neurons, NeuroDevSim run times varied between 19.5 min for serial simulation and 0.84 min for 32-core simulation (Figure 9A). Start-up times were negligible at 0.08 s for serial simulation, increasing to 0.10 s for 32-core simulation. This compares to much slower NeuroMaC run times for the same model, varying between 39.5 h for serial simulation and 36.0 min for 32 core simulations (Figure 9D) with a close to constant start-up time of approximately 2 s. In conclusion, NeuroDevSim simulations are approximately two orders of magnitude faster than NeuroMaC simulations (Figure 9E), confirming the speed-ups obtained from using shared memory parallel simulation with continuous scheduling of front processing compared to a messaging-based spatial parallelization.

How effective is the parallelization? This was evaluated by computing the strong scaling, where linear performance corresponds to a reduction of run time by a factor of two for doubling the number of processing cores. As shown in Figure 9B, NeuroDevSim achieves close to optimal scaling for up to eight cores, but for 16 and 32 cores, there is reduced scalability. Scalability depends on the size of the model simulated, with a smaller loss of scalability for the 200-neuron forest than for the 50- or 100-neuron forests. This dependence on model size indicates that the scaling problem is caused at the end of each simulation cycle when an increasing number of processing cores become idle while the last few fronts are processed by a subset of cores. The total simulation run times scale close to linear with forest size: for the 50-neuron forest, 9.2 min for serial simulation and 0.43 min for 32 cores, and 39.2 and 1.65 min for the 200-neuron forest, respectively. The more effective strong scaling for the larger forest (Figure 9B) is therefore due to the idle time at the end of each simulation cycle becoming shorter relative to the duration of the cycle. Using hyperthreading to simulate with 64 processing cores was not effective (not shown); this was expected because the physical cores are kept quite busy.

The results of Figures 8A, 9B show an effect of model size on the effectiveness of parallelization. This is confirmed by analyzing the strong scaling of the cerebellar model of Kato and De Schutter (2023) which simulates approximately 800,000 fronts and adds front migration and retraction to the growth simulated in the previous tests (Figure 9C). During the initial part of the cerebellar simulation, until cycle 70, only granule cell migration and parallel fiber extensions are simulated, achieving an optimal parallel scaling for up to 96 processing cores. Later, when the import_simulation call and retractions of Purkinje dendrites that operate in serial mode are added, parallel scaling is affected but still reaches an optimum at 96 processing cores.

Another factor affecting scaling for a large number of cores might have been delays during _lock_broker calls (Figures 6, 7). Because _lock_broker is run intermittently on the Admin_agent core, there may be short wait times before it responds and this might scale with the number of processing cores. Therefore, a separate series of benchmarks was run where _lock_broker ran continuously on a separate core. This did not result in any speed-up (not shown), and using an extra core was not an effective parallelization strategy. Similarly, using two separate admin cores, one for recording output and one for scheduling, did not lead to a significant speed-up.

Finally, strong scaling was also evaluated for NeuroMaC (Figure 9D). Here, performance was initially supra-linear, with a big speed increase when going from serial to parallel with just two processing cores. But this effect only lasted up to 16 processing cores, with comparatively little gain for 32 cores.

### Effect of grid parameters and GridCompetionError

To perform efficient collision detection for new fronts, only nearby existing fronts should be tested for overlap. Nearby fronts are found by mapping all fronts to a grid (Figure 5), with a default grid_step distance of 20 μm between _grid points and storage of front IDs to linked _grid_extra blocks of 10 items. The distance between _grid points should be matched to the average size of fronts so that, ideally, each front is allocated to a single _grid point. The effect of changing grid_step was evaluated for the 100-neuron forest simulation (Table 1). Front lengths in this model vary between 3.5 and 8 μm, with most of them being 7 μm long. This suggests that the default grid_step is too large. Reducing it to 10 μm indeed improved run times, but a further decrease to 5 μm was less efficient. Decreasing grid_step led to an increase in the mean number of _grid points used for each front but led to a smaller number of fronts attached to each occupied _grid point. The latter, with, for example, 99% occupied _grid points having only 1 to 10 fronts for grid_step = 10, strongly affects the size of the collision detection search. Because 10 is the default size of _grid_extra blocks, the effect of this parameter was also tested. Increasing block size to 20 or 30 entries improved the mean run time for the single compute core simulations to 1164 and 1117 s, respectively, but gave no systematic improvements for multi-core simulations.

**TABLE 1:** The effect of grid_step size on _grid use and run times for the 100-neuron forest simulation (*n* = 10).

grid_step:	5 μm	10 μm	20 μm (default)	30 μm
Mean number of _grid points used/front	1.98	1.53	1.29	1.19–1.20
Occupancy of _grid	0.3%	1.0–1.1%	3.2–3.4%	5.9–6.2%
Fraction occupied _grid points with 1–5 fronts	99%	88–90%	46–48%	33–34%
Fraction occupied _grid points with 1–10 fronts	100%	99%	79–81%	58–60%
Largest number of fronts per _grid point	10	18–21	54–73	96–133
Mean run time 1 processing core (sec)	1099	1035	1172	n.a.
Mean run time 32 processing cores (sec)	50.2	43.6	44.6	n.a.

Either a mean value or a range is provided unless the measure is constant for all simulations. As grid_step becomes smaller, the _grid array increases in size, and less of it is used, leading to reduced occupancy. The simulations generated 129,179 to 145,606 fronts.

**FIGURE 10:**
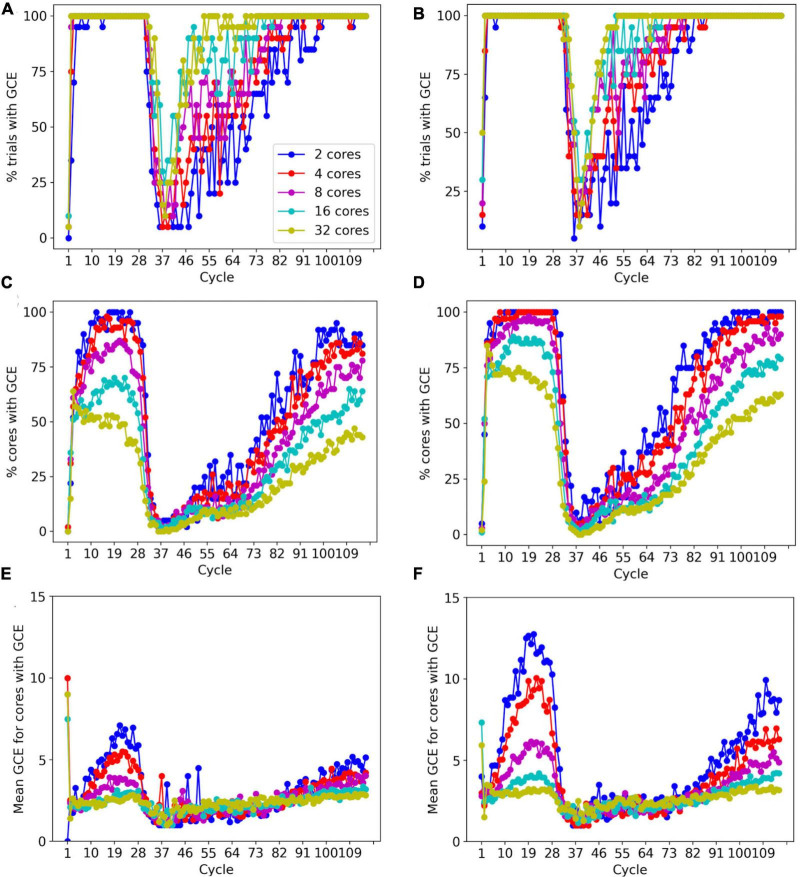
Effect of the number of cores used on GridCompetitionError (GCE) during different simulation cycles. Simulations of a 100-neuron L5 pyramidal forest model [left, **(A,C,E)**] or a 200-neuron model [right, **(B,D,F)**] (*n* = 20). **(A,B)** Percentage of trials that had a GridCompetitionError vs. the simulation cycle for the number of processing cores shown in the legend in **(A)**. **(C,D)** Percentage of cores that had a GridCompetitionError vs. the simulation cycle for the number of processing cores shown in the legend in **(A)**. **(E,F)** Mean number GridCompetitionErrors for each core vs. simulation cycle for the number of processing cores shown in the legend in **(A)**.

To prevent memory corruption, access to the _grid is locked (Figures 6, 7). If a core cannot access a locked _grid entry for a long time, it will return the method call with a GridCompetitionError. The occurrence of GridCompetitionError at different cycles of the forest simulation was investigated (Figure 10). The number of GridCompetitionError varied strongly, with peaks both at the beginning and end of the simulation. This reflects the stages of growth of the basal and tuft dendrites, respectively, while during the mid-stage, the apical and oblique dendrites grow. Basal and tuft dendrites grow in relatively crowded environments and therefore different cores will try to access overlapping _grid areas more often, resulting in more errors. Comparing GridCompetitionError for a forest of 100 neurons (Figures 10A, C, E) with one of 200 neurons (Figures 10B, D, F) confirms the crowding effect as more errors were generated in the 200-neuron forest (Figures 10E, F). The relation with the number of processing cores used is more complex. As expected, the fraction of trials where GridCompetitionError occurs increased with the number of cores used (Figures 10A, B); the effect is most obvious during the mid-stage. This can be explained by the challenge Admin_agent has to avoid scheduling overlapping regions in the simulation volume to different cores at each cycle when the number of cores is large. However, when analyzing either the fraction of cores that experienced a GridCompetitionError (Figures 10C, D) or the mean number of errors per core (Figures 10E, F), the numbers were systematically larger for simulations with fewer processing cores. This is because for a given number of GridCompetitionError, the fraction per core will always be larger for few than for many cores. The total number of GridCompetitionError remained higher for simulations with many cores; e.g., in Figure 10F, at cycle 20, the total number of GridCompetitionError was 24 for 2 cores and 68 for 32 cores.

The results in Figure 10 show that GridCompetitionError is an unavoidable side effect of how grid locking is implemented, but when handled properly in code, it does not prevent very effective parallelization (Figure 9). Neither does it affect the size of the neurons grown, with the number of fronts generated in each simulation being highly variable (Figure 8B) but covering similar ranges for 16 and 32 computing cores.

## Discussion

This paper describes NeuroDevSim, a simulator of neural development that can model the migration of neurons, growth of dendrites and axons, and formation of early synaptic connections. In this regard, NeuroDevSim replicates most of the functionality of the no longer maintained CX3D (Zubler et al., 2013) with the exception of progenitor cell proliferation. Though the birth of new neurons can be made conditional in NeuroDevSim by adding the relevant statements before the add_neurons method (Figure 2), modeling actual neural proliferation is a goal for future NeuroDevSim versions. In practice, the simulation of neural proliferation is rather artificial in a fixed simulation volume as used in both CX3D and NeuroDevSim. Brain regions undergoing large cell proliferation rapidly expand in volume [e.g., (Legue et al., 2015)] and not simulating this expansion leads to artificially increased crowding conditions in the model. In our simulation of cerebellar development, this was identified as a major limitation of the model (Kato and De Schutter, 2023). Nevertheless, as shown in Zubler et al. (2013); Torben-Nielsen and De Schutter (2014), and Kato and De Schutter (2023) highly detailed models of neural tissue development can be simulated with these simulators.

Besides enabling the simulation of neural development, NeuroDevSim also introduces an innovative way of parallelizing computation based solely on using shared memory in multi-core architectures. The number of cores on common CPUs has increased rapidly and our approach can now be used with up to 128 cores on platforms that combine two 64-core AMD CPUs (Figure 9C). Because there are no communication delays in shared memory simulation, it outperforms messaging-based parallelization, spectacularly as demonstrated in the comparison of NeuroDevSim to NeuroMaC run times (minutes vs. hours in Figure 9) and achieves optimal parallelization for up to 96 cores for the Kato and De Schutter (2023) model without front retractions. As it is likely that future CPUs will offer increasing numbers of cores, NeuroDevSim can be expected to simulate even larger and more complex models in the future. The performance can be further improved by fine-tuning simulator parameters like grid_step (Figure 5 and Table 1).

The linear strong scaling of NeuroDevSim over model-dependent ranges (Figure 9) is better than the consistent sublinear strong scaling achieved by BioDynaMo (Breitwieser et al., 2022), which uses threading-based parallelization. Nevertheless, BioDynaMo claims to run a model of the growth of 5000 pyramidal neurons in 35 s on 72 cores, which would make it approximately eight times faster than NeuroDevSim based on a linear extrapolation from the 200-neuron forest data. This can be explained by multiple factors. The C++ implementation of BioDynaMo is faster than Python software like NeuroDevSim. The agents used by BioDynaMo to simulate pyramidal neurons have a much simpler code than the NeuroDevSim ones, omitting layer dependency and lacking repulsion between tuft dendrites. Finally, though BioDynaMo uses a similar cartesian grid, it is less rigorous in its collision avoidance than NeuroDevSim by not checking for collisions among agents instantiated during the same cycle.

The implementation of shared memory parallelization introduces unique programming challenges: the use of fixed-size data arrays and the need to avoid memory write conflicts. Though not explored in this study, we expect that these challenges are fairly easy to solve for fixed data size problems. The size of data arrays is predictable for such applications and the use of private sections of data arrays for writing as demonstrated in Figures 3, 4 should be sufficient to avoid memory conflicts. Unfortunately, this did not apply to NeuroDevSim because array sizes are not predictable when modeling stochastic growth. As a consequence, array sizes need to be estimated and, in practice, this is often based on trial and error. If NeuroDevSim models become too large for the default array size parameters, the simulation will crash with an OverflowError that mentions which array size parameter needs to be increased. In the absence of OverflowError, the allocated arrays are often too large (Table 1), but with CPU memory use in the range of 200 MB to 2 GB, this should not be a concern on most computing platforms.

With this caveat, most data allocation and updating could be handled in a rigorous way that avoids memory conflicts, except for collision detection. Collision avoidance is a tough challenge in parallel computing, irrespective of whether messaging or shared memory approaches are used. Solving the conflict of new structures that overlap in physical space being generated on different cores requires, by definition, the interaction between the computing cores involved. In the case of messaging-based parallelization, this requires first communication to detect a possible collision and when a collision occurs, it requires extra messaging to solve it; the latter may be challenging if more than two cores are involved. In the case of our implementation of shared memory parallelization (Figure 5), detection is fast, but solving the collision is less tractable because cores do not communicate directly. Therefore, the simulator tries to avoid these problems as much as possible by not scheduling simultaneous updating of fronts that occupy nearby areas of the simulation space. This approach works quite well as evidenced by the improved strong scaling for larger models compared to smaller ones (Figure 9) despite the increasing number of possible collisions. But this algorithm is not perfect, and situations can occur where irreconcilable locking conflicts happen. In such a case the method returns a GridCompetitionError. Analysis (Figure 10) shows, as expected, that this will occur more frequently when many cores are used but it will happen even for a small number of cores. Therefore, the user needs to deal with GridCompetitionError in the model code; the simplest solution is usually to try again a few times, and if that fails, repeat it during the next cycle.

In fact, besides these challenges, shared memory computation requires the software user to follow a set of strict rules that are outlined in the NeuroDevSim documentation. Unusual for Python programs, instance attributes cannot be used and many front or synapse attributes should never be accessed or changed directly; instead, specific methods have to be called. Similarly, fronts or synapses can only be made using NeuroDevSim methods; they cannot be instantiated by the user. As already mentioned, the user may have to change preset array sizes during the Admin_agent instantiation. Many coding examples are provided to help users learn how to deal with these challenges, and once one gets used to the specific coding requirements, NeuroDevSim models can be written in a clear and concise style.

NeuroDevSim 1.0 is the first version of the software. As we gain more experience in using it to simulate real development, additional features and improvements will be added. Current plans include additional options for axon growth during soma migration and automated tracking of pioneer axons. To mimic dendritic development more closely, the continued growth of dendritic diameters will be enabled. More challenging is solving the artificial crowding caused by the fixed simulation volume. One solution is to enable the simulation of tissue expansion, but this requires quite a change of modeling style. Whereas currently neurons are created in an empty space and initially grow without collisions, tissue expansion assumes starting with a fully packed model where growth is achieved by pushing other structures aside. Implementing this at a phenomenological level, using simple geometries like spheres and cylinders and without computing forces in detail, will require extensive investigation.

## Data availability statement

The datasets presented in this study can be found in online repositories. The names of the repository/repositories and accession number(s) can be found below: https://github.com/CNS-OIST/NeuroDevSim.

## Author contributions

ED developed the software, tested it, and wrote the manuscript.

## Footnotes

1
https://www.sqlite.org/

2
http://geomalgorithms.com/

3
https://zeromq.org

4
https://en.wikipedia.org/wiki/Linked_list
